# Nitric Oxide Production from Nitrite Reduction and Hydroxylamine Oxidation by Copper-containing Dissimilatory Nitrite Reductase (NirK) from the Aerobic Ammonia-oxidizing Archaeon, *Nitrososphaera viennensis*

**DOI:** 10.1264/jsme2.ME18058

**Published:** 2018-10-12

**Authors:** Shun Kobayashi, Daisuke Hira, Keitaro Yoshida, Masanori Toyofuku, Yosuke Shida, Wataru Ogasawara, Takashi Yamaguchi, Nobuo Araki, Mamoru Oshiki

**Affiliations:** 1 Department of Civil Engineering, National Institute of Technology Nagaoka College, Nagaoka Japan; 2 Department of Applied Life Science, Faculty of Biotechnology and Life Science, Sojo University Ikeda, Kumamoto Japan; 3 Graduate School of Life and Environmental Sciences, University of Tsukuba Tsukuba, Ibaraki Japan; 4 Department of Bioengineering, Nagaoka University of Technology Nagaoka, Niigata Japan; 5 Department of Science of Technology Innovation, Nagaoka University of Technology Nagaoka Japan

**Keywords:** nitrite reduction, hydroxylamine oxidation, nitrous oxide production, ammonia oxidizing archaea, *Nitrososphaera viennensis*

## Abstract

Aerobic ammonia-oxidizing archaea (AOA) play a crucial role in the global nitrogen cycle by oxidizing ammonia to nitrite, and nitric oxide (NO) is a key intermediate in AOA for sustaining aerobic ammonia oxidation activity. We herein heterologously expressed the NO-forming, copper-containing, dissimilatory nitrite reductase (NirK) from *Nitrososphaera viennensis* and investigated its enzymatic properties. The recombinant protein catalyzed the reduction of ^15^NO_2_^−^ to ^15^NO, the oxidation of hydroxylamine (^15^NH_2_OH) to ^15^NO, and the production of ^14–15^N_2_O from ^15^NH_2_OH and ^14^NO_2_^−^. To the best of our knowledge, the present study is the first to document the enzymatic properties of AOA NirK.

Aerobic ammonia oxidation, a rate-limiting step of nitrification, drives the global nitrogen cycle (24, 40), which involves aerobic ammonia-oxidizing archaea and bacteria (AOA and AOB, respectively) and complete ammonia oxidizers (comammox) (9, 44). Of these, AOA primarily contribute to aerobic ammonia oxidation in natural environments including soil and open ocean (19, 31, 46). AOA are affiliated with the phylum *Thaumarchaeota*, which includes phylogenetically and physiologically diverse members (6) and the soil-inhabiting archaeon *Nitrososphaera viennensis* (41). The biochemistry of aerobic ammonia oxidation by AOA has received a great deal of interest because ammonia oxidation to nitrite (NO_2_^−^) proceeds in a different manner to that of AOB. AOA oxidize ammonia to hydroxylamine by ammonia monooxygenase (Amo) as well as AOB (43), while hydroxylamine is further oxidized to NO_2_^−^ by an unidentified enzyme (17). All known AOA genomes lack the gene encoding hydroxylamine dehydrogenase (Hao), and the involvement of a copper-protein complex has been proposed (40, 45). In parallel with the oxidation of ammonia to NO_2_^−^, AOA produce nitric oxide (NO) (22). NO is a key intermediate in AOA cells because this highly reactive molecule is essential for sustaining aerobic ammonia oxidation activity (17, 33, 36, 47). To date, the following 2 pathways have been reported as a source of prokaryotic NO formation: NO_2_^−^ reduction to NO by copper-containing and cytochrome *cd_1_*-type dissimilatory nitrite reductases (NirK and NirS, respectively) (38) and NH_2_OH oxidation to NO by hydroxylamine oxidoreductase (Hao) (4, 21). Although neither *nirS* nor *hao* are found in AOA genomes (6), AOA commonly possess *nirK*, which is transcribed and expressed during aerobic ammonia oxidation (8, 15, 20, 37). These findings suggest that NirK are involved in NO formation in AOA cells. However, NO_2_^−^ reduction to NO by AOA NirK has never been demonstrated.

Bacterial NirK have been characterized as homotrimeric enzymes, and each subunit has 2 Cu-binding sites (Type 1 and 2 Cu-binding sites). Type 1 Cu-binding sites receive an electron from an electron donor, such as type 1 Cu proteins (single-domain cupredoxins) and/or cytochrome *c*, and the electron is then further transferred to a type 2 Cu-binding site that is the catalytic center of NirK (14, 25). Bacterial NirK have been classified into 2 phylogenetically distinct groups (class 1 and class 2 groups) based on sequence similarities, and the NirK of the class 1 group contains linker loop and tower loop regions in the amino acid sequence (3). AOA NirK, including *Ns. viennensis* NirK, are affiliated with a distinct clade of bacterial class 1 and 2 groups (Fig. 1A). Lund *et al.* (20) reported that AOA NirK may be further classified into several phylogenetic clades showing specific geographic distributions. *Ns. viennensis* NirK has amino acid residues consistent with those of type 1 and 2 Cu-binding sites (His_106_, His_140_, and His_316_ for type 1 Cu-binding sites and His_101_, Cys_141_, His_152_, and Met_157_ for type 2 Cu-binding sites) as well as the linker and tower loop regions, whereas the C terminus has unusual extensions of ~26 residues (Fig. 1B). These phylogenetic affiliations of and structural variations in *Ns. viennensis* NirK raise concerns regarding its enzymatic properties, such as specific enzymatic activity, affinity for NO_2_^−^, and products of NO_2_^−^ reduction.

Based on its unique sequence and lack of biochemical information, the purpose of the present study was to characterize *Ns. viennensis* NirK. Prior to the present study, we aimed to isolate *Ns. viennensis* NirK from a batch culture of *Ns. viennensis* as a native enzyme. However, the activity of aerobic ammonia oxidation often disappeared when we scaled up the cultures (data not shown). Additionally, a slow growth rate (*μ_max_* 0.024 h^−1^) (41) and low biomass concentration in the culture (*ca.* 10^7~8^ cells mL^−1^) further precluded the preparation of the biomass required for protein purification. Since recombinant NirK proteins have been successfully used to previously examine several enzymatic properties (7, 16, 32), the authors decided to heterologously express *Ns. viennensis* NirK in *Escherichia coli*, and investigate its enzymatic properties. The *nirK* gene located in the *Ns. viennensis* genome (accession number; CP007536.1) was cloned into the expression vector pCold I (Takara Bio, Shiga, Japan) with the 6×His tag using the Mighty cloning reagent set (Takara Bio), and transformed into *E. coli* strain BL21(DE3) (Takara Bio). The N-terminal region of *Ns. viennensis* NirK was predicted to be the signal peptide sequence (Met_1_ to Ala_24_), and *nir*K without the signal peptide sequence was amplified by PCR using ExTaq polymerase (Takara Bio) and specific forward (5′-GGCATATGGCCCCGACTGGTGTCACTAGACACTAT-3′) and reverse (5′-GGAAGCTTAACCAGAGGTGGTGTTGC CACCGGAGG-3′) oligonucleotide primers. The restriction sites of NdeI and HindIII in the forward and reverse primers above are underlined. Genomic DNA extracted from *Ns. viennensis* cells (JCM19564) was used as the DNA template for PCR. The constructed plasmid was subjected to Sanger sequencing, and no mutations were found in the sequence. Regarding the expression of the recombinant protein in *E. coli* cells, the expression culture was aerobically cultivated at 37°C in Luria-Bertani media containing 100 ng μL^−1^ ampicillin. When the OD_600_ of the culture increased to 0.4, the culture was transferred to 15°C and held for 30 min, and protein expression was then induced by adding isopropyl β-D-1-thiogalactopyranoside (IPTG) at a final concentration of 0.1 mM. After being incubated at 15°C for 24 h, cells were harvested by centrifugation at 8,500×*g* at 4°C for 10 min. The harvested cells were suspended in buffer containing 20 mM Tris HCl (pH 8), 200 mM NaCl, and 10% glycerol. The cells were disrupted using a sonifier 250 (Branson) (output 20, duty 20% for 60 s, 6 cycles), and centrifuged at 13,000×*g* at 4°C for 1 h. The supernatant was recovered as a soluble protein fraction, and the recombinant protein was purified using His-tag affinity chromatography. The recombinant protein was bound to His60 Ni Superflow resin (Takara Bio), and washed with washing buffer containing 20 mM Tris HCl (pH 8), 200 mM NaCl, 10% glycerol, and 20 mM imidazole. The bound recombinant protein was eluted with elution buffer containing 20 mM Tris HCl (pH 8), 200 mM NaCl, 10% glycerol, and 300 mM imidazole. Protein concentrations were measured using the DC-protein assay kit (Bio-Rad, Hercules, CA, USA) with bovine serum albumin as previously described (26), and purity was evaluated by sodium dodecyl sulfate-polyacrylamide gel electrophoresis (SDS-PAGE) on a 10% polyacrylamide gel as previously described (28). As shown in Fig. 2A, a single protein band appeared at a molecular mass of 40 kDa, which closely matched the molecular mass deduced from amino acid sequences of the recombinant protein (*i.e.*, 39.7 kDa). The protein band was excised from the gel, and subjected to a matrix-assisted laser desorption ionization-time of flight mass spectrometry (MALDI-TOF MS) analysis after in-gel tryptic digestion for protein identification (The detailed methodology is described in the Supplementary text). The MALDI-TOF MS analysis confirmed that the protein band corresponded to *Ns. viennensis* NirK (Fig. S1). Regarding the reconstitution of Cu-binding sites of the recombinant protein, the purified recombinant protein was dialyzed against buffer containing 20 mM Tris HCl (pH 8), 300 mM NaCl, and 0.5 mM CuSO_4_ at 4°C for 57 h. The protein solution was dialyzed again using the above Tris buffer without CuSO_4_ at 4°C for 6 h. The dialyzed recombinant protein was concentrated using a Vivaspin column (MWCO; 30 kDa) (GE Healthcare Japan, Tokyo, Japan). The recombinant protein was loaded onto a gel-filtration HiLoad 16/600 Superdex 200 pg column (GE Healthcare) to assess the molecular mass of the recombinant protein, which was 105±1.3 kDa (Fig. 2B). Since the deduced molecular mass of *Ns. viennensis* NirK was 39.7 kDa, the molecular mass obtained by gel filtration indicated that the recombinant protein forms a homotrimeric structure, similar to canonical NirK.

NirK have been characterized as metalloproteins showing a blue or green color spectrum, and exhibit absorption peaks at approximately 450 and/or 600 nm (3). Bacterial NirK, which belong to the class 1 group, often show a maximum absorption peak at approximately 450 nm, although an exception (*Achromobacter xylosoxidans* NirK) that shows a peak at 593 nm has been previously reported (16). The purified recombinant protein was pale blue in color, and showed an absorption peak at 590 nm (Fig. 2C). This feature indicated that *Ns. viennensis* NirK is affiliated with the subgroup of NirK showing a blue color spectrum. The blue or green color spectrum of NirK is derived from a copper atom in the type 1 Cu-binding site (14), while the type 2 Cu-binding site does not contribute to the UV or visible spectrum. The type 2 Cu-binding site shows a characteristic electron spin resonance (ESR) spectrum (7, 16); therefore, an ESR analysis was performed using a JES-FA200 spectrometer (JEOL, Tokyo, Japan) to test for the presence of the type 2 Cu-binding site in the recombinant protein. An axial type 2 Cu signal (g_//_=2.24, A_//_=18.31 mT, and *g*_⊥_=2.06) was found in the ESR measurement (Fig. 2D), indicating that the recombinant protein has a type 2 Cu-binding site coordinating with a copper atom. Additionally, we assessed the copper content of the recombinant protein by inductively coupled plasma mass spectrometry (ICP-MS). The copper content was found to be 2.9 atoms per subunit of the recombinant protein, indicating that Cu was fully incorporated into the recombinant protein. Overall, the recombinant protein shared the structural and spectroscopic features of class 1 and 2 bacterial NirK, which is consistent with sequencing information.

The kinetics of NO_2_^−^ reduction were examined by anoxically incubating the recombinant protein at 25°C and pH 6.5 with ^15^NO_2_^−^ and artificial electron donors as previously described (7). All of the buffers and stock solutions were prepared anoxically as previously described (27). Two milliliters of reaction buffer (20 mM phosphate buffer, 0.1 to 1.6 mM Na^15^NO_2_^−^, 0.5 mM benzyl viologen (BV), and 0.24 mM sodium dithionite) was dispensed into a 1-cm sealable quartz cuvette and placed in an anaerobic chamber in which the O_2_ concentration was maintained at lower than 1 ppm. BV was used as an artificial electron donor because it has been employed to examine the kinetics of the NO_2_^−^ reduction of bacterial NirK (7, 13). The cuvette was set in a UV-VIS spectrometer UV-2700 (Shimadzu, Kyoto, Japan), and the initial absorbance of the prepared reaction mixture at a wavelength of 550 nm was approximately 2.0. The reaction was initiated by adding the recombinant protein (50 μL containing 250 μg of protein) using a gastight syringe, and the oxidation rate of reduced BV (molecular extinction coefficient, 10.4 mM^−1^ cm^−1^) (13) was monitored at 550 nm. The recombinant protein reduced NO_2_^−^ by oxidizing BV, whereas no significant BV oxidation was found in the cuvette without the recombinant protein. The turnover number and *K_m_* value for NO_2_^−^ reduction by the recombinant protein were 3.1 s^−1^ and 287 μM, respectively (Table 1), and the turnover number and affinity constant were markedly lower and higher, respectively, than those of other canonical NirK proteins, including those from AOB. The product of NO_2_^−^ reduction by the recombinant protein was examined using phenazine methosulfate (PMS) as the electron donor instead of BV. When BV was used as the electron donor, NO_2_^−^ was reduced to NO, and further reduced to ammonia (approx. 60% of consumed ^15^NO_2_^−^) as observed in a previous study in which the NO_2_^−^ reduction activity of *A. xylosoxidans* NirK was examined using methyl viologen (MV) as the electron donor (1). BV and MV have low redox potentials (−350 and −440 mV, respectively) (23), resulting in the reduction of NO to NH_3_; therefore, PMS with a higher redox potential (+80 mV) was used in the present study. The recombinant protein was incubated as described above in a 1.8-mL gas-tight vial with the addition of 0.5 mM PMS and 5 mM ascorbic acid instead of BV and dithionite, and the production of ^15^N-labeled gaseous compounds (*i.e.*, N_2_, NO, and N_2_O) in the headspace was examined by gas chromatography mass spectrometry (GC/MS) as previously described (27). The diluted gases of ^15-15^N_2_ (Cambridge Isotope Laboratories, Tewksbury, MA, USA), ^14^NO, and ^14-14^N_2_O (GL Science, Tokyo, Japan) were also analyzed to prepare standard curves for quantification. The recombinant protein reduced ^15^NO_2_^−^ with the oxidation of PMS, and 38 and 48% of consumed ^15^NO_2_^−^ were converted to ^15^NO and ^15-15^N_2_O, respectively. This is direct evidence to show that the recombinant protein is a NO-forming nitrite reductase. We found that the production of ^15-15^N_2_O was equal to the production of ^15^NO, which likely results from the reduction of ^15^NO_2_^−^ to H^15^NO (*i.e.*, NO_2_^−^+2e^−^+3H^+^ → HNO+H_2_O) and the chemical formation of ^15-15^N_2_O from the formed H^15^NO (*i.e.*, 2HNO → N_2_O+H_2_O) (35), as previously observed for a sulfide-linked nitrite reductase (34).

Aside from NO_2_^−^ reduction, NH_2_OH oxidation was also investigated using the recombinant protein because NH_2_OH is produced as an intermediate during aerobic ammonia oxidation by AOA. The kinetics of NH_2_OH oxidation were examined by aerobically incubating the recombinant protein (245 μg mL^−1^) at 30°C and pH 7.5 with 0.5 mM NH_2_OH, with dissolved oxygen being available as an oxidant. The reaction was initiated by the addition of NH_2_OH solution, and the concentration of NH_2_OH was assessed colorimetrically (5). The concentration of H_2_O_2_, which may be produced by the oxidase activity of NirK (12), was also evaluated colorimetrically using horseradish peroxidase (Wako, Osaka, Japan) and 3,3′,5,5′-tetramethylbenzidine (TMBZ) (Dojindo, Kumamoto, Japan) (2). As shown in Fig. S2, the recombinant protein oxidized NH_2_OH with the production of H_2_O_2_. No NH_2_OH oxidation or H_2_O_2_ production was observed when the incubation was repeated without the addition of the recombinant protein. The values for the turnover number and affinity constant for NH_2_OH oxidation were 0.039 s^−1^ and 97 μM (Table 1), respectively, and the value for the turnover number was two orders of magnitude lower than that observed for NO_2_^−^ reduction; therefore, the recombinant protein catalyzed NO_2_^−^ reduction more efficiently. The addition of cytochrome *c* from equine heart (1 mg mL^−1^) or BV (0.5 mM) did not result in an increase in the reaction rate or affinity for NH_2_OH oxidation. The product of NH_2_OH oxidation by the recombinant protein was examined in a ^15^NH_2_OH tracer experiment (29). The recombinant protein was incubated in a 1.8-mL gas-tight vial with the addition of 0.5 mM ^15^NH_2_OH (Cambridge Isotope Laboratories) instead of ^14^NH_2_OH. After a 2-h incubation, the concentrations of the ^15^N-labeled gaseous products were assessed by GC/MS. The recombinant protein oxidized ^15^NH_2_OH and produced ^15^NO, ^15-15^N_2_O, and ^15-15^N_2_ gases quantitatively (Fig. 3), whereas the production of NO_2_^−^ and NH_3_ was not detectable (detection limits: 50 and 100 μM, respectively). The oxidation of NH_2_OH to NO has been described in bacterial Hao (21); however, to the best of our knowledge, this is the first description of NH_2_OH oxidation by NirK. We also observed ^15-15^N_2_O production from ^15^NH_2_OH oxidation, which likely resulted from the oxidation of ^15^NH_2_OH to H^15^NO and abiotic coupling of H^15^NO, as previously described. Notably, ^15-15^N_2_ was the major product of ^15^NH_2_OH oxidation by the recombinant protein. Hydroxylamine disproportionation (30) may not be responsible for ^15-15^N_2_ production because NH_3_ production was not detectable in the liquid phase. The molecular mechanisms underlying the oxidation of ^15^NH_2_OH to ^15-15^N_2_ by the recombinant protein warrant further studies.

We repeated the above incubation with the addition of NH_2_OH and NO_2_^−^ because both compounds are available in AOA cells during aerobic ammonia oxidation. Therefore, the above incubation was repeated with the addition of ^15^NH_2_OH and ^14^NO_2_^−^ (each 0.5 mM) or ^14^NH_2_OH and ^15^NO_2_^−^ (Cambridge Isotope Laboratories) (each 0.5 mM). In both cases, ^14–15^N_2_O was the major product (Fig. 3), indicating that the recombinant protein produces N_2_O by oxidizing NH_2_OH using NO_2_^−^ as an electron acceptor. N_2_O production by the denitrifier NirK from NH_2_OH and NO_2_^−^ has been previously described (10), and the N-nitrosation reaction is involved in N_2_O production (39). Notably, *Ns. viennensis* cells produce N_2_O when they are incubated aerobically with NH_3_ and NO_2_^−^ (42), although the *Ns. viennensis* genome lacks the gene encoding nitric oxide reductase (*nor*) that is involved in N_2_O production from nitrifier-denitrification. Stieglmeier *et al.* (42) suggested the involvement of *Ns. viennensis* NirK in the production of N_2_O in an *Ns. viennensis* culture, and our results support this hypothesis. Although the catalytic efficiency of *Ns. viennensis* NirK for NH_2_OH oxidation was markedly lower than that of NO_2_^−^ reduction (Table 1), *Ns. viennensis* NirK may act as an NH_2_OH oxidase in *Ns. viennensis* cells and produce N_2_O under oxic growth conditions. Aside from ^14–15^N_2_O production, the production of ^15^NO and ^15-15^N_2_O was also observed when the recombinant protein was incubated with ^14^NH_2_OH and ^15^NO_2_^−^ (Fig. 3).

Although the recombinant protein catalyzes NO_2_^−^ reduction and NH_2_OH oxidation, the catalytic efficiency of both reactions was low, as shown in Table 1. AOA *nirK* transcripts are abundant in the transcriptome (8, 11, 20, 37), suggesting the strong expression of AOA NirK in cells. NirK was the 225^th^ most abundant protein of the 1,503 proteins detected in the proteome of the late exponential phase of *Ns. viennensis* cells aerobically oxidizing ammonia (15). The strong expression of NirK appears to support the activity of NO_2_^−^ reduction to NO as well as NH_2_OH oxidation to NO by the low efficiency catalytic enzyme. *Ns. viennensis* NirK may function as a bifunctional enzyme that supplies NO molecules from 2 different sources (*i.e.*, NH_2_OH and NO_2_^−^), which provides *Ns. viennensis* cells with a competitive advantage. In the present study, the enzymatic kinetics of recombinant *Ns. viennensis* NirK for NO_2_^−^ reduction were examined using artificial electron donors; further studies are needed to identify physiological electron donors in *Ns. viennensis* cells. Bacterial NirK may accept electrons supplied from single-domain cupredoxin and cytochrome *c* (14, 25). A number of genes encoding single-domain cupredoxin were found in the *Ns. viennensis* genome (Table S1), whereas the ortholog of the gene encoding cytochrome *c* was not. To date, the biochemistry of AOA cupredoxin has not been investigated using natural enzymes and recombinant proteins, and our study provides basic information that furthers our understanding of the biochemistry of AOA.

## Supplementary text



## Figures and Tables

**Fig. 1:**
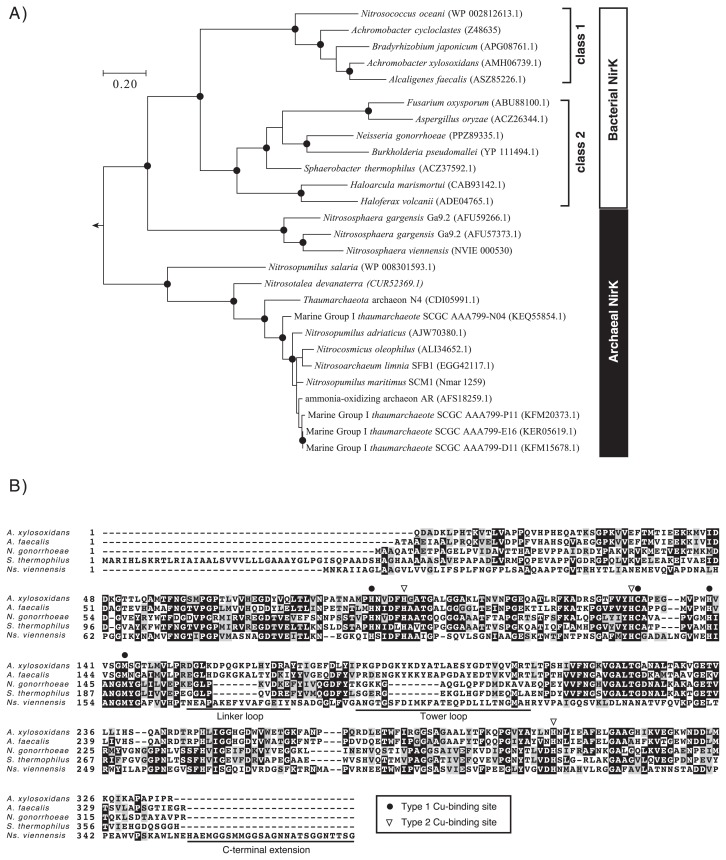
Phylogeny (A) and sequence alignments (B) of prokaryotic NirK. A) A phylogenetic tree of prokaryotic NirK was constructed by the maximum likelihood method with the Jones-Taylor-Thornton model using the protein sequence of multicopper oxidase type 3 of *Nitrososphaera viennensis* (accession number; AIC14243.1) as an outgroup. Branching points that support a probability >80% in bootstrap analyses (based on 500 replicates) are shown as filled circles. The scale bar represents 10% sequence divergence. Sequence accession numbers are indicated in parentheses. B) Protein sequence alignment of *nirK*. NirK sequences were aligned using ClustalW software. Circles and triangles correspond to the amino acid residues of type 1 and 2 Cu-binding sites, respectively. Linker, Tower loop (3), and C-terminal extension regions are underlined. Abbreviations of microorganisms are as follows: *Nitrosomonas europaea* is *N. europaea*, *A. xylosoxidans* is *Achromobacter xylosoxidans*, *A. faecalis* is *Alcaligenes faecalis*, *N. gonorrhoeae* is *Neisseria gonorrhoeae*, *S. thermophilus* is *Sphaerobacter thermophilus*, and *Ns. viennensis* is *Nitrososphaera viennensis*.

**Fig. 2:**
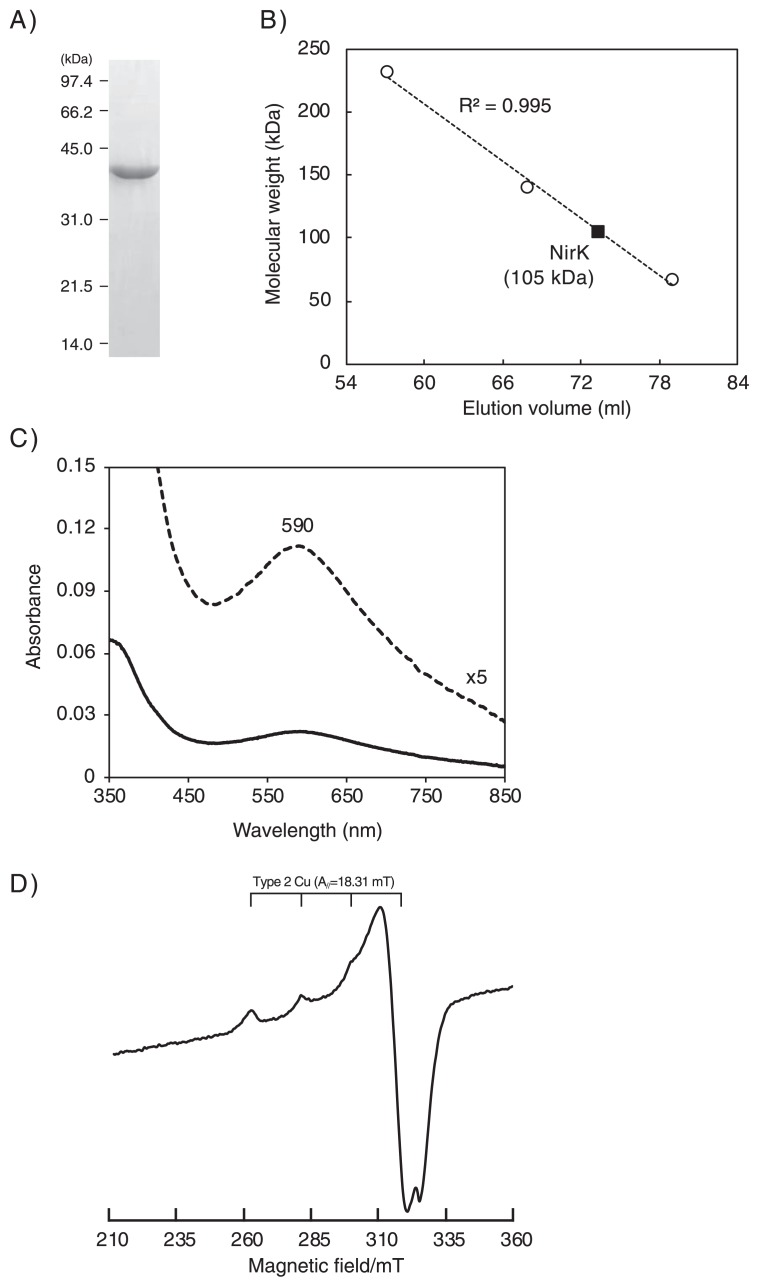
Characterization of recombinant *Nitrososphaera viennensis* NirK. A) SDS-PAGE of the recombinant protein purified by His-tag affinity chromatography. B) Assessment of the molecular mass of the recombinant protein by gel filtration chromatography. Catalase from bovine liver (232 kDa), lactate dehydrogenase (140 kDa), and bovine serum albumin (66 kDa) were used to prepare a standard calibration curve. C) UV-VIS absorption spectra. The measurement was performed in a 20 mM Tris buffer (pH 8) containing 300 mM NaCl at 25°C. The solid line indicates the recombinant protein (1 mL mL^−1^) oxidized with air. A 5×enlarged spectrum is also shown as a dashed line. D) ESR spectra. The measurement was performed using the recombinant protein (4.9 mg mL^−1^) at −253°C.

**Fig. 3:**
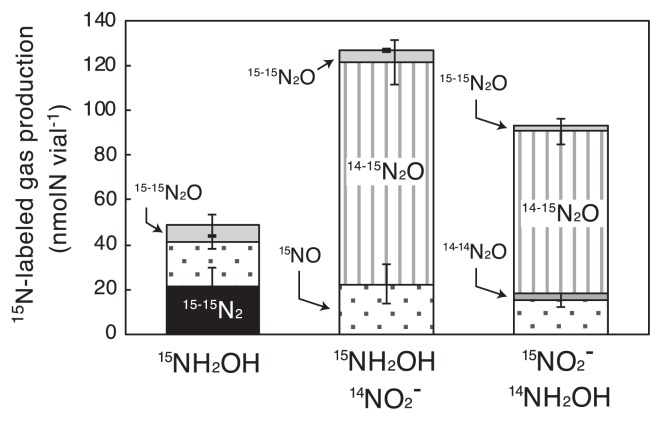
NH_2_OH oxidation by recombinant *Nitrososphaera viennensis* NirK. The recombinant protein was incubated at 30°C and pH 7.5 in 1.8-mL vials (volume of the headspace: 1.5 mL), with i) 0.5 mM ^15^NH_2_OH, ii) ^15^NH_2_OH and ^14^NO_2_^−^ (each 0.5 mM), or iii) ^14^NH_2_OH and ^15^NO_2_^−^. The production of N_2_, NO, and N_2_O in the headspace was examined by gas chromatography mass spectrometry (GC/MS). NH_3_ and NO_2_^−^ concentrations were also measured; however, they were not detectable during the incubation. During a 2-h incubation, i) 63±35 (mean±SD), ii) 149±1, and iii) 120±1 nmol N of NH_2_OH were consumed in the liquid phase, resulting in 75–137% of the ^15^N-labeled nitrogen mass balance in the vials. Error bars represent the SD derived from triplicate incubations, and the graph bars represent the mean values. NH_2_OH oxidation was not found in the vials without the addition of the recombinant protein.

**Table 1:** Enzymatic properties of archaeal and bacterial copper-containing nitrite reductase (NirK). ND; not determined.

Organisms	MW* (kDa)	Cu content† (atom per subunit)	Absorption (nm)	Activity‡ Turnover (s^−1^)	*K_m_* (μM)	Reference
Archaeal NirK						
* Nitrososphaera viennensis*	105±1.5	2.9	590			
NO_2_^−^ reduction				3.1	287	This study
NH_2_OH oxidation				0.039	97	This study
Bacterial NirK (NO_2_^−^ reduction)						
* Nitrosomonas europaea*	96	ND	450, 597	288	ND	18
* Nitrosococcus oceani*	114	1.67	455, 575	1,600	52	16
* Achromobacter xylosoxidans*	110	1.99	595	172	35	14, 32
* Candidatus* Jettenia caeni	101	ND	449, 598	319	250	7

* Molecular weight (MW) of a trimeric NirK. The MW of *Ca.* Jettenia caeni NirK was calculated from amino acid sequences without a signal peptide sequence.

† Copper contents previously assessed by chemical analyses were shown.

‡ The following electron donors were used to evaluate the turnover number of NO_2_^−^ reduction; methyl viologen for *N. europaea* and *Nc. oceani*, pseudoazurine for *A. xylosoxidans*, and benzyl viologen for *Ns. viennensis* and *Ca.* Jettenia caeni NirK.
