# Supplementary material for: Nitric Oxide Production from Nitrite Reduction and Hydroxylamine Oxidation by Copper-containing Dissimilatory Nitrite Reductase (NirK) from the Aerobic Ammonia-oxidizing Archaeon, Nitrososphaera viennensis
Source: Microbes Environ. 2018 Oct 12;33(4):428–34. doi: 10.1264/jsme2.ME18058 (PMC6308003; doi:10.1264/jsme2.ME18058)
Supplement: Supplementary file 1 [file 33_428_s1.pdf]

Supplementary text

Nitric oxide production from nitrite reduction and hydroxylamine oxidation by copper-containing dissimilatory nitrite reductase (NirK) from the aerobic ammonia-oxidizing archaeon, *Nitrososphaera viennensis*

Shun Kobayashi[1], Daisuke Hira[2], Keitaro Yoshida[3], Masanori Toyofuku[3], Yosuke Shida[4], Wataru Ogasawara[4], Takashi Yamaguchi[5], Nobuo Araki[1] & Mamoru Oshiki[1*]

[1]Department of Civil Engineering, National Institute of Technology, Nagaoka College, Nagaoka, Japan

[2]Department of Applied Life Science, Faculty of Biotechnology and Life Science, Sojo University, Ikeda, Kumamoto, Japan

[3]Graduate School of Life and Environmental Sciences, University of Tsukuba, Tsukuba, Ibaraki, Japan

[4]Department of Bioengineering, Nagaoka University of Technology, Nagaoka, Niigata, Japan

[5]Department of Science of Technology Innovation, Nagaoka University of Technology, Nagaoka, Japan.

[*]Corresponding author:

Mamoru Oshiki (Ph.D.)

E-mail; oshiki@nagaoka-ct.ac.jp

Tel/Fax; +81-258-34-9277/9284

Experimental procedures

in-gel tryptic digestion and matrix assisted laser desorption ionization-time of flight mass spectrometry (MALDI-TOF MS) analysis

Once excised from a polyacrylamide gel, the gel band was first destained using a destaining buffer that contained 25 mM NH_4HCO_3 and 30% (v/v) acetonitrile. The proteins in the destained gel band were reduced by incubation in 10 mM dithiothreitol for 20 min at 56°C, and then alkylated by incubation in 50 mM iodoacetamide for 45 min at 20°C under dark conditions. Trypsin digestion was performed by incubating the gel band overnight at 37°C in 50 mM ammonium bicarbonate buffer, containing 25 ng μL^{-1} of trypsin (Trypsin Gold, Mass Spectrometry Grade) (Promega, Tokyo, Japan). The digested peptides were sequentially extracted by incubating for 30 min in 1% (w/v) trifluoroacetic acid (TFA) buffer and 100% acetonitrile. The recovered peptide solutions were pooled and spotted on a metal MALDI-TOF sample plate using ZipTip C_{18} pipette tips. Conditioning of the ZipTip C_{18} pipette tip and desalting of the peptide solution with the ZipTip C_{18} pipette tip were performed according to the manufacturer's instructions. An equal volume of the MALDI matrix solution, containing 50% (v/v) acetonitrile, 0.05% TFA, and 10 mg mL^{-1} α-cyano-4-hydroxycinnamic acid (Tokyo Chemical Industry, Tokyo, Japan), was directly mixed with the spotted peptide solution and dried. Mass spectrometric analysis was performed in the mass range of 800-4,000 Da on a MALDI-TOF MS autoflex III mass spectrometer, using a MALDI calibration standard that contained a 6-peptide mixture (Protea Biosciences, Morgantown, WV, USA). The determined peptide masses were subjected to a MASCOT search. The amino acid sequences of the gene coding sequences (CDSs), located in the *Nitrososphaera viennensis* genome, were used as the reference database, and the search parameters were as follows: fixed modification of carbamidomethyl (C), variable modification of oxidation (M), ± 1.2 Da peptide mass deviation, and one miscleavage was permitted.

Table S1. Genes encoding copper-containing proteins of *Nitrososphaera viennensis*. Orthologs were investigated by a blastP search (blastP, threshold *e*-value; 10^{-10}). Amino acid sequences of genes located in the *Ns. viennensis* genome (accession number; CP007536.1) were used as query sequences, and indicated with locus_tag numbers in the table. Amino acid sequences of bacterial and archaeal cupredoxins were retrieved from the RefSeq database (2,918 sequences in total), and used as reference sequences of the blastP search.

Query	product	best blastP hit in the RefSeq database	identity	*e*-value
NVIE_002600	putative blue (type1) copper domain-containing protein	WP_046120229.1 plastocyanin [*Sinorhizobium* sp. PC2]	35.87	2.00E-14
NVIE_003910	putative cupredoxin	WP_078657213.1 plastocyanin [*Kitasatospora aureofaciens*]	38.27	5.00E-12
NVIE_005340	putative copper-binding, plastocyanin/azurin family protein	WP_014496806.1 amicyanin [*Bradyrhizobium japonicum*]	35.88	1.00E-15
NVIE_011200	hypothetical protein	WP_081438107.1 plastocyanin [*Frankia asymbiotica*]	40	2.00E-13
NVIE_011230	hypothetical protein	WP_046120229.1 plastocyanin [*Sinorhizobium* sp. PC2]	48.65	2.00E-23
NVIE_014370	putative copper-binding protein, plastocyanin/azurin family	WP_024517370.1 amicyanin [*Bradyrhizobium* sp. Tv2a-2]	39.17	6.00E-15
NVIE_020060	exported protein of unknown function	WP_007167886.1 MULTISPECIES:_amidase [*Mycobacterium*]	32.87	5.00E-13
NVIE_023620	blue (type 1) copper domain protein	WP_014496806.1 amicyanin [*Bradyrhizobium japonicum*]	34.56	5.00E-16
NVIE_024190	protein of unknown function with a C-terminal blue (Type 1) copper domain	WP_017294320.1 plastocyanin [*Geminocystis herdmanii*]	40.48	2.00E-14
NVIE_027530	putative heme-copper oxidase subunit II	WP_028457668.1 cytochrome c oxidase subunit II [*Chloroflexus* sp. Y-396-1]	34.88	1.00E-12
NVIE_029580	blue (Type 1) copper domain-containing	WP_038168083.1 plastocyanin	40.24	7.00E-13

Query	product	best blastP hit in the RefSeq database	identity	*e*-value
	protein	[*Thiomonas* sp. FB-Cd]		

Supporting figure legends

Fig. S1. Identification of recombinant *Nitrososphaera viennensis* NirK by a MASCOT search. The protein sequences highlighted in red correspond to the peptide masses obtained by a matrix-assisted laser desorption ionization-time of flight mass spectrometry (MALDI-TOF MS) analysis, and the significant identification of *N. viennensis* NirK was supported by the probability-based Mowse score. The signal peptide sequence of *N. viennensis* NirK (*i.e.*, Met_1 to Ala_{24}) is not described in the figure because recombinant NirK, without the signal peptide sequence, was expressed in the present study.

Fig. S2. Time-course measurements of NH_2OH oxidation by recombinant *Nitrososphaera viennensis* NirK. The recombinant protein was incubated at 30°C and pH 7.5 with 0.5 mM $^{14}NH_2OH$. The concentrations of NH_2OH and H_2O_2 (closed and open symbols, respectively) were assessed colorimetrically. The incubation was also performed without the recombinant protein in parallel, and no NH_2OH consumption or H_2O_2 production was observed. Error bars represent standard deviations derived from quadruplicate incubations, and the graph symbols represent mean value.

APTGVTRHYTLIANEMEVQVAPDNALHPGGIKYNAMVFNGTIPGPVMASNAGDTVEITLKNEGKQIH
SIDFHAAIGPSQVLSGNIAAGESKTWTFNTPNSGAFMYHCGADALNGVWEHIANGMYGAFVVHPTN
EAPAKEFYVAFGEIYNSADGGLFVGANGTGSFDIMKFATEQPDLILTNGMAHRYVPAIGQSVKLDLN
ANATVFQVKPGELTRWYILAPGPNEGVSFHFISGQIDVRDGSFKTRNMAPVRNEETWWIPVGSASVI
ESVFPEEGLYVGVDHNMAHVLRGGAFAVLATNNSTADDVPPEAWVPSKAWLNEHAEMGGSMMG
GSAGNNATSGGNTTSG

Fig. S1 (Kobayashi et al.)

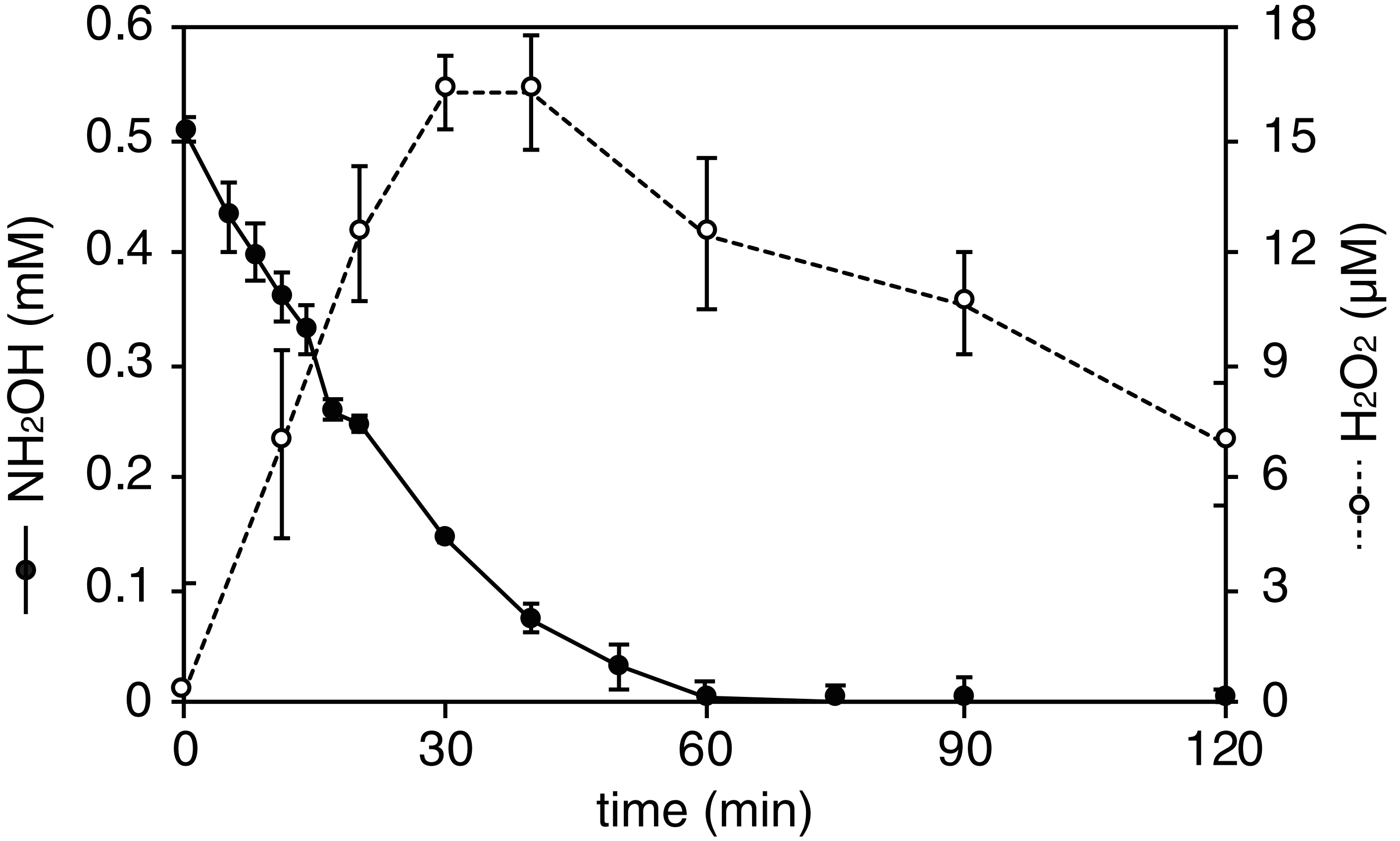

Fig. S2 (Kobayashi et al.)
